# Évaluation de la Transmission de la Filariose Lymphatique au Burkina Faso: À Propos de 4 Districts Sanitaires

**DOI:** 10.48327/mtsibulletin.n1.2021.83

**Published:** 2021-04-04

**Authors:** A. Kima, K.T. Guiguemde, M. Serme, Z.C. Meda, R. Bougma, J.P. Djiatsa, C. Bougouma, F. Drabo

**Affiliations:** 1 Programme national de lutte contre les maladies tropicales négligées, Ouagadougou, Burkina Faso; 2 Laboratoire de parasitologie UFR-SDS, Université Ouaga 1 Joseph KI-ZERBO, Ouagadougou, Burkina Faso; 3 Institut supérieur des sciences de la santé, Université NAZI BONI, Bobo Dioulasso, Burkina Faso; 4 ONG Helen Keller International, Ouagadougou, Burkina Faso

**Keywords:** Filariose lymphatique, Traitement médicamenteux de masse, Impact, *Wuchereria bancrofti*, Léo, Sapouy, Boromo, Dédougou, Burkina Faso, Afrique subsaharienne, Lymphatic filariasis, Mass drug administration, Impact, *Wuchereria bancrofti*, Leo, Sapouy, Boromo, Dedougou, Burkina Faso, Sub-Saharan Africa

## Abstract

**Objectif:**

Dans le cadre d'une étude d'impact de la chimiothérapie préventive contre la filariose lymphatique au Burkina Faso, le Programme national de lutte contre les maladies tropicales négligées (PNMTN) a conduit une enquête d'évaluation de la transmission dans les districts sanitaires de Léo, Sapouy, Boromo et Dédougou. L'objectif principal était d'évaluer le niveau de transmission de la filariose lymphatique dans ces 4 districts sanitaires répartis en 2 unités d'évaluation (UE) (Boucle du Mouhoun 3 et du Centre-Ouest 2) après au moins 9 ans de traitement médicamenteux de masse.

**Méthodologie:**

Il s'est agi d'une enquête transversale à visée descriptive réalisée en milieu communautaire sur un échantillon d'enfants de 6 à 7 ans sélectionnés par sondage en grappe.

**Résultats:**

Sur 1649 enfants enquêtés dans l'UE Centre-Ouest 2, quatre étaient positifs au Filariasis Test Strip (FTS), soit une proportion de porteurs d'antigène filarien circulant (signant la présence de stades adultes vivant de *Wuchereria bancrofti* ) de 0,24%. Dans l'UE BMH3, aucun des 1716 enfants enquêtés n'était positif au FTS.

**Conclusion:**

Ces résultats montrent que la transmission de la filariose lymphatique est interrompue dans ces districts sanitaires, où les traitements de masse à l'albendazole et l'ivermectine peuvent donc être interrompus.

## Abstract (en)

**Objective:**

In this impact survey on the preventive chemotherapy against lymphatic filariasis, the national neglected tropical diseases programme team conducted a Transmission Assessment Survey in the health districts of Leo, Sapouy, Boromo and Dedougou. The purpose of this study was to assess lymphatic filariasis transmission in these four districts (included in two evaluation units (EU): Boucle du Mouhoun 3 and Centre-Ouest 2 after more than ten to thirteen years of mass drug treatment.

**Methodology:**

The study was a cross sectional survey which targeted the school aged children based on the cluster survey method conducted at community level.

**Results:**

Among the 1649 school aged children covered by the survey in the Centre Ouest EU, four were found positive at the Filariasis Test Strip (FTS), i.e. the proportion of children with circulating filarial antigens (i.e. with live adult stages of *W. bancrofti* ) was 0.24%. In the BMH3 EU, none of the 1716 children tested was FTS-positive.

**Conclusion:**

From the findings, we can infer that filariasis transmission has been interrupted in these districts and that mass treatment with albendazole and ivermectin can be stopped.

## Introduction

La filariose lymphatique (FL) est une maladie tropicale négligée (MTN) à transmission vectorielle. A l'instar d'autres MTN, la FL affecte les populations pauvres rurales et marginalisées [6]. Une enquête menée en 2000 au Burkina Faso, avant toute intervention de contrôle, avait montré que dans certains villages, la proportion de sujets présentant des antigènes filariens circulants (détecté par le test rapid immunochromatographic, ICT) atteignait 74%, indiquant que le pays était sévèrement touché par la FL à *Wuchereria bancrofti* [8]. Soutenues par des partenaires techniques et financiers et l'engagement de l'État, des campagnes de chimiothérapie préventive basées sur le traitement combiné par albendazole et ivermectine ont été lancées en 2001. Celles-ci ont permis de modifier considérablement le profil épidémiologique de la FL dans plusieurs districts sanitaires (DS) du pays dont ceux de Léo et Sapouy dans la région sanitaire du centre-ouest (C-O) et ceux de Dédougou et Boromo dans la région de la boucle du Mouhoun (BMH). Au Burkina Faso, la mise en oeuvre de ces campagnes se fait sous la coordination du Programme national de lutte contre les MTN (PNMTN). Les traitements médicamenteux de masse (TMM) sont annuels dans la plupart des DS endémiques pour la FL et semestriels dans cinq DS de la région du sud-ouest. En 2015, la chimiothérapie contre la FL a touché 5393933 personnes vivant dans 30 DS endémiques.

La surveillance de l'impact des TMM sur la FL comprend plusieurs phases et cette activité est organisée au sein de zones géographiques appelées « unités d'évaluation » (UE). Chaque UE peut comprendre plusieurs DS. Quand les populations ont reçu cinq TMM, avec des couvertures thérapeutiques considérées comme satisfaisantes (c'est-à-dire ≥65%), une première enquête peut être organisée dans des sites sentinelles (définis une fois pour toute au début du processus) et des sites de contrôle ponctuels (qui peuvent varier entre les phases) pour évaluer la proportion de sujets de 5 ans et plus présentant des antigènes filariens circulants (donc des vers adultes vivants) ou des microfilaires sanguines. Si la prévalence de l'antigénémie est inférieure à 2% dans tous les sites sélectionnés dans l'UE ou si la prévalence de la microfilarémie est inférieure à 1% dans tous ces sites, une enquête d'évaluation de la transmission (*Transmission Assessment Survey*, TAS) ciblant les enfants peut être organisée pour déterminer si l'on peut arrêter les TMM.

De 2005 à 2015, les couvertures thérapeutiques relevées dans l'UE Centre-Ouest 2 (C-O2) variaient, en fonction des années, de 78,6 à 85,4%. Dans l'UE Boucle du Mouhoun 3 (BMH3), elles variaient de 75,7 à 83,0% [7, 15].

En 2015, les résultats des enquêtes « Pré-TAS » montraient que les prévalences de la microfilarémie dans les populations des sites sentinelles et de contrôle étaient inférieures à 1% dans les deux UE (respectivement 0,2% et 0,4% pour l'UE C-O2 et 0% et 0,2% pour l'UE BMH3). L'objectif de cet article est de présenter les résultats de l'enquête TAS réalisée en 2016 dans les quatre DS mentionnés plus haut, après plus de neuf ans de TMM à l'albendazole et l'ivermectine.

## Méthodes

### Type et cadre d'étude

**Fig. 1:**
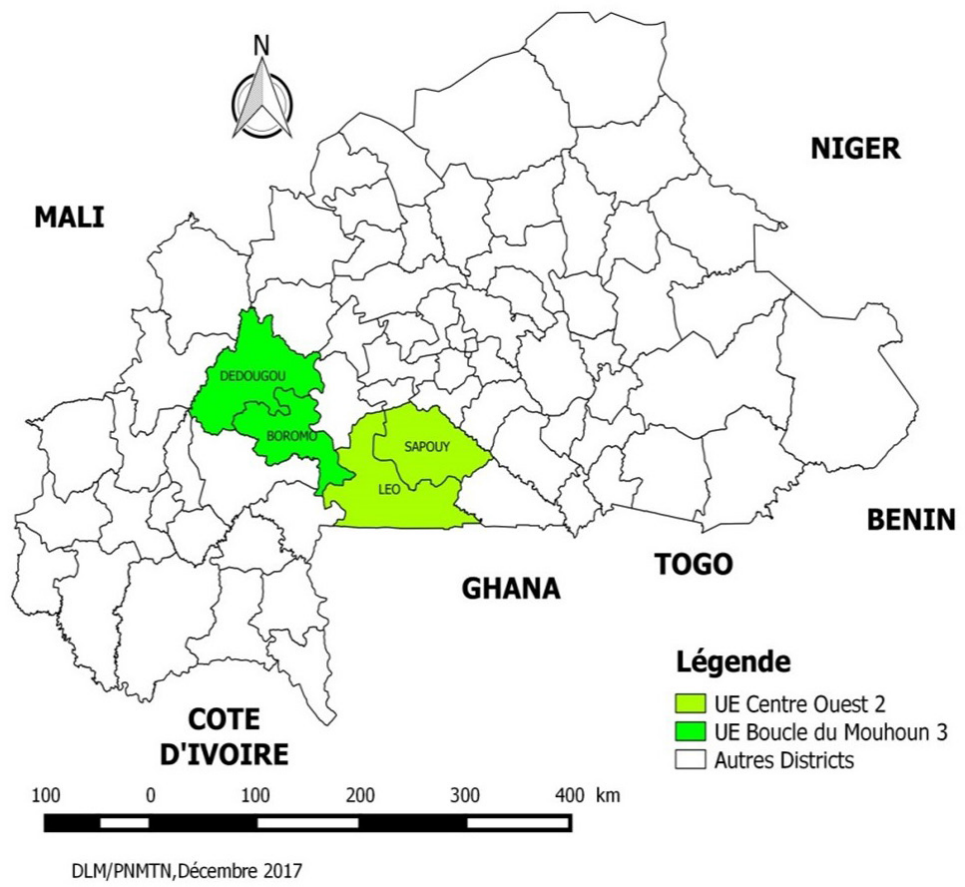
Carte du Burkina Faso montrant les districts étudiés Map of Burkina Faso showing the study districts

L'évaluation de la transmission a été réalisée par une enquête transversale descriptive communautaire menée du 9 au 24 août 2016 auprès d'un échantillon de ménages de l'UE C-O2 constituée des DS de Léo et Sapouy et de l'UE BMH3 constituée des DS de Dédougou et Boromo (Fig. 1). Le climat de la zone d'étude est de type sahélo-soudanien caractérisé par une saison sèche et une saison pluvieuse.

### Population d'étude

La population incluse dans l'étude était représentée par les enfants âgés de 6 à 7 ans des quatre DS. Ce choix repose sur l'hypothèse que si le TMM communautaire depuis plus de 5 ans a été efficace pour interrompre la transmission, ces enfants devraient être protégés contre l'infestation filarienne.

### Méthode d'échantillonnage

Le nombre d'enfants cibles automatiquement généré par le logiciel Microsoft Excel Survey Sample Builder (SSB) était de 1684 pour l'UE BMH3 et de 1556 pour l'UE C-O2.

**Fig. 2:**
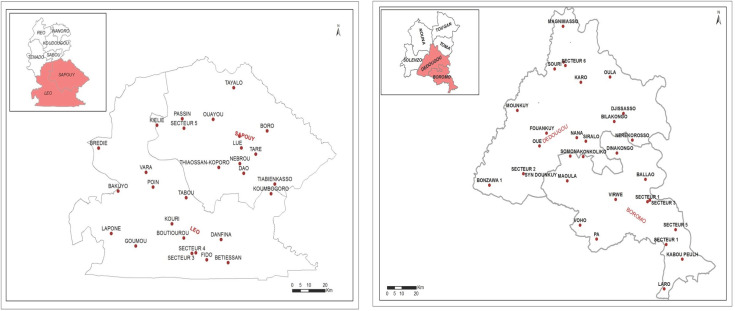
Localisation des clusters étudiés lors des enquêtes TAS pour la FL au Burkina Faso en 2016 Location of studied clusters surveyed during TAS surveys for LF in Burkina Faso in 2016

Au regard du faible taux net de scolarisation, inférieur à 75% dans les UE ciblées, l'enquête a concerné les enfants de 6 et 7 ans au sein de la communauté conformément aux directives [10, 12]. Il s'est agi d'un sondage en grappe à deux degrés (premier degré: grappes ou zone de dénombrement (ZD), deuxième degré: ménages). La sélection de ces grappes s'est faite de façon aléatoire à l'aide du logiciel SSB à partir de la liste des ZD établies lors du recensement général de la population et de l'habitation (RGPH) de 2006 [9]. Ainsi, 30 grappes ont été générées pour chaque UE, soit 60 pour les 2 UE (Fig. 2). Une liste complémentaire de cinq grappes était prévue pour compléter l'échantillon au cas où la taille n'était pas atteinte. Le nombre d'enfants attendu par grappe était de 53 enfants dans l'UE BMH3 et de 52 enfants pour l'UE C-O2. Un dénombrement des ménages dans chacune des grappes a fourni une liste des ménages à partir de laquelle a été tiré un échantillon sur la base de deux listes de nombres aléatoires générés par le logiciel SSB [11, 12]. Etaient considérés comme éligibles tous les enfants de 6 et 7 ans vivant dans le ménage sélectionné et résidant depuis au moins six mois dans l'UE. Dans chaque ménage sélectionné la recherche des cas d'hydrocèle et de lymphoedème était systématique.

### Recueil des données

La collecte des données a été réalisée après entretien individuel à l'aide d'une fiche d'enquête adaptée du questionnaire du manuel de référence de 2011 de l'OMS [12].

Un échantillon de 75 microlitres de sang capillaire a été recueilli dans un tube capillaire hépariné. L'échantillon était placé sur la bandelette FTS (Filariasis Test Strip) qui est un outil de diagnostic rapide pour la détection d'antigènes filariens circulants dont la présence signe la présence de stades adultes vivants de *W. bancrofti*. La lecture du résultat a été faite exactement 10 minutes après le dépôt de l'échantillon de sang, comme recommandé par le fabricant. Un résultat positif se traduit par l'apparition de deux traits roses sur la bandelette.

### Analyse des données

Les données ont été saisies et analysées au moyen des logiciels Excel et Epi Info version 7.2.

La valeur seuil est le seuil de prévalence de l'infestation en dessous duquel on estime qu'une transmission ne peut pas être entretenue et qu'une recrudescence est peu probable, même en l'absence de TMM. Le seuil critique automatiquement généré par l'outil SSB était de 20 cas positifs pour UE BMH3 et 18 cas positifs pour UE C-O2 [16].

Un nombre total de cas positifs égal ou inférieur au seuil critique dans une UE indique qu'il n'y a pas de transmission [12].

### Considérations éthiques

Le protocole de cette étude a été approuvé par le comité éthique de recherche du Ministère de la santé. Une fois sur le terrain, les équipes de collecte des données ont clairement expliqué, en langue locale, aux autorités dirigeantes du village et aux parents des enfants, le protocole et les objectifs de l'étude. Un consentement éclairé écrit a été obtenu des parents des enfants retenus pour l'enquête.

## Résultat

### Proportion d'enfants présentant des antigènes filariens circulants

Au total, 1649 enfants de 6 à 7 ans dont 868 de sexe masculin (53%) ont été enquêtés dans l'UE C-O2. Le nombre moyen d'enfants enquêtés par grappe était de 55. Les enfants de 6 ans étaient prédominants avec 847 (51%).

Sur les 1649 enfants testés, quatre, dont trois de sexe masculin, étaient positifs au FTS soit une prévalence de l'antigénémie de 0,24% [Intervalle de confiance (IC): 0,1-0,7].

Dans l'UE BMH3, 1716 enfants dont 881 de sexe masculin (51%) ont été enquêtés. Le nombre moyen d'enfants enquêtés par grappe était de 54. Les enfants de 7 ans étaient prédominants (886, soit 52%). Aucun enfant n'était FTS positif soit une prévalence de l'antigénémie de 0% (Tableau I).

**Tableau I:** Nombre d'enfants examinés et nombre et proportion d'enfants positifs au FTS dans les deux UE enquêtées Number of children screened and number and proportion of FTS positive children in the two EUs surveyed

UE	Pop totale 2016	Nombre de grappes enquêtées	Nombre d'enfants examinés	Seuil critique	Positif à FTS	Prévalence de l'antigénémie
**Centre-Ouest 2**	526 592	30	1649	18	4	0,24%
**Boucle du Mouhoun 3**	670 528	32	1716	20	0	0%

FTS: Filariasis Test Stri

### Évaluation des morbidités

Le nombre de cas d'hydrocèle estimé dans les ménages enquêtés était de 14 et 18 respectivement dans les UE C-O2 et BMH3. Pour les cas de lymphoedèmes des membres inférieurs, le nombre estimé était de 2 et 7 respectivement dans les UE C-O2 et BMH 3. Ces cas de morbidité étaient retrouvés uniquement chez les adultes.

## Discussion

La présente évaluation a permis de collecter des données sur un grand échantillon représentatif. Cependant, il a fallu recourir à 32 grappes dans l'UE BMH3 au lieu de 30 prévues pour atteindre la taille d'échantillon requise. Le faible taux de scolarisation n'a pas permis de réaliser l'enquête en milieu scolaire. L'enquête s'est déroulée pendant la saison hivernale en milieu communautaire et des difficultés liées à la mobilisation des enfants étaient fréquentes dans certaines grappes. Pendant la saison hivernale, certaines grappes étaient difficilement accessibles par les enquêteurs. En effet, le nombre d'enfants de 6 et 7 ans obtenu dans les 30 grappes de l'UE BMH3 n'atteignait pas la taille de l'échantillon attendue générée par le logiciel SSB.

L'outil de diagnostic utilisé lors de cette évaluation était un outil rapide et bien accepté, recommandé par l'OMS et dont les performances ont par ailleurs été bien documentées [20].

Le nombre d'enfants trouvés positifs au FTS (4 et 0) était très inférieur aux valeurs seuils critiques de 18 et 20, respectivement, pour les UE C-O2 et BMH 3 témoignant ainsi l'impact favorable de plus de 9 années de TMM dans les deux UE enquêtées.

Les résultats de la présente étude témoignent sans équivoque de l'absence de transmission de la filariose lymphatique dans les 4 DS et de la possibilité d'y arrêter le traitement sans risque de recrudescence. Ces résultats viennent porter à 45 le nombre de DS ayant déjà arrêté le TMM sur un ensemble de 70 DS que compte le Burkina Faso.

La prévalence de *W. bancrofti* au Burkina Faso était parmi les plus élevées en Afrique, comme en témoignent les résultats des travaux de Brengues en 1975 [3] et ceux de la cartographie réalisée en 2000. Au Burkina Faso tout comme dans les autres pays de l'Afrique de l'Ouest les vecteurs principaux sont *Anopheles gambiense* et *An. funestus* [1, 4, 13].

A l'instar d'autres régions sanitaires du Burkina Faso, les régions du Centre-Ouest et de la Boucle du Mouhoun étaient classées zones endémiques avec des prévalences d'antigénémie de 4,9 à 48% pour la BMH 3 et de 32 à 58% pour le Centre-Ouest 2 selon les données épidémiologiques de base recueillies en 2000 [8]. Ces prévalences comparées à celles de 2016 montrent un changement favorable du profil épidémiologique de la filariose lymphatique dans ces régions. Cette situation laisse croire que l'élimination de la FL est possible au Burkina Faso à l'instar d'autres pays comme le Togo et le Malawi, le Japon, la Chine, la République de Corée et les Iles Salomon [14, 21].

Ces résultats sont le fruit de la pertinence et de l'efficacité des stratégies mises en place par le PNMTN, de la détermination des acteurs de terrain et du soutien continu des partenaires techniques et financiers, sans occulter l'adhésion des communautés au traitement de masse.

Dans le souci d'empêcher la réémergence de la maladie dans la communauté, la lutte antivectorielle par l'utilisation intensive et régulière de moustiquaires imprégnées d'insecticide à longue durée d'action s'impose car son efficacité dans le contrôle de la filariose lymphatique a été prouvée par des études récentes [2, 17, 18].

En outre, un rapprochement entre les services de santé, de l'éducation et de l'environnement permettra d'asseoir une durabilité des acquis déjà engrangés dans le contrôle de cette maladie.

Au cours de cette étude, 32 cas d'hydrocèle et 9 cas de lymphoedème des membres inférieurs ont été retrouvés chez les adultes dans les deux UE. Au regard des pertes de productivité annuelles liées aux incapacités dues à la filariose [5, 19], un modèle épidémiologique plus approprié permettrait de faire des estimations plus fiables des cas de lymphoedème et d'hydrocèle afin de mieux planifier la mise en oeuvre de la prise en charge des morbidités.

La prise en charge des morbidités en tant que deuxième stratégie majeure de lutte contre la filariose lymphatique est toujours peu développée. L'évaluation de la transmission de la filariose lymphatique dans la communauté constitue une opportunité pour recenser les cas de morbidité dus à la FL notamment les cas d'hydrocèle et de lymphoedème.

## Conclusion

Les résultats de cette étude sont en faveur d'un arrêt des traitements de masse contre la filariose lymphatique dans les DS de Léo, Sapouy, Boromo et Dédougou. Cependant, il convient de mettre en place un système efficace de surveillance épidémiologique post-arrêt TMM afin de détecter une éventuelle recrudescence de la transmission. Aussi, une investigation autour des cas trouvés positifs pourrait permettre la détection de foyers résiduels de transmission du fait que la FL est également une maladie focale.

## Remerciements

La réussite de cette étude découle d'une large collaboration de toutes les personnes impliquées. Le Programme national de lutte contre les maladies tropicales négligées remercie les partenaires techniques et financiers (USAID/Helen Keller International) dont l'engagement et les appuis ont rendu possible la mise en oeuvre de cette évaluation. Enfin, une motion spéciale de remerciement est adressée aux enquêteurs, superviseurs, aux personnes enquêtées et aux populations des villages enquêtés.

## Conflits D'intérêts

Les auteurs ne déclarent aucun conflit d'intérêts.
